# Uncommon Adverse Events of Immune Checkpoint Inhibitors in Small Cell Lung Cancer: A Systematic Review of Case Reports

**DOI:** 10.3390/cancers16101896

**Published:** 2024-05-16

**Authors:** Eunso Lee, Jeong Yun Jang, Jinho Yang

**Affiliations:** 1 Division of Allergy and Pulmonology, Department of Internal Medicine, Chungnam National University Sejong Hospital, Sejong 30099, Republic of Korea; 2 Department of Radiation Oncology, Konkuk University Medical Center, Konkuk University School of Medicine, 120-1, Neungdong-ro, Gwangjin-gu, Seoul 05030, Republic of Korea; 3 Department of Occupational Health and Safety, Semyung University, 65 Semyung-ro, Jecheon 27136, Republic of Korea

**Keywords:** small cell lung cancer, immune checkpoint inhibitor, adverse event, case reports, systematic review

## Abstract

**Simple Summary:**

This study systematically reviewed case reports to identify rare adverse events in small cell lung cancer (SCLC) patients treated with immune checkpoint inhibitors (ICIs). A literature search, limited to reports published up to 31 December 2023, yielded 24 studies involving various ICIs, including atezolizumab, durvalumab, and nivolumab, either as monotherapy or in combination treatments. The review documented adverse events predominantly affecting the respiratory system, along with neurological, endocrinological, and gastroenterological systems. A total of 87.5% of cases showed symptom improvement, although some reports indicated severe outcomes, including death or persistent sequelae. The findings highlight the unpredictability of ICIs’ adverse effects and emphasize the importance of developing reliable biomarkers and multidisciplinary strategies to enhance patient management and safety in immunotherapy.

**Abstract:**

Background: This study aimed to systematically review case reports documenting rare adverse events in patients with small cell lung cancer (SCLC) following the administration of immune checkpoint inhibitors (ICIs). Methods: A systematic literature review was conducted to identify case reports detailing previously unreported adverse drug reactions to ICIs in patients with SCLC. The scope of the literature reviewed was restricted to case studies on SCLC published up to 31 December 2023. Results: We analyzed twenty-four studies on ICI use for patients with SCLC. There were six reports on atezolizumab, four on durvalumab, and three on adverse events from monotherapy with nivolumab. Reports involving combination treatments were the most frequent, with a total of six, predominantly involving using nivolumab in combination with ipilimumab. Additionally, there was one report each on using pembrolizumab, nofazinilimab, sintilimab, tislelizumab, and toripalimab. We collected detailed information on the clinical course, including patient and disease characteristics, symptoms, treatment for each adverse event, and recovery status. Among the patients included in the case reports, 21 out of 24 (87.5%) had extensive-stage SCLC when initiating ICI therapy, with only 1 patient diagnosed with limited-stage SCLC. Respiratory system adverse events were most common, with seven cases, followed by neurological, endocrinological, and gastroenterological events. Three case reports documented adverse events across multiple systems in a single patient. In most cases, patients showed symptom improvement; however, four studies reported cases where patients either expired without symptom improvement or experienced sequelae. Conclusions: Efforts to develop reliable biomarkers for predicting irAEs continue, with ongoing research to enhance predictive precision. Immunotherapy presents diverse and unpredictable adverse events, underscoring the need for advanced diagnostic tools and a multidisciplinary approach to improve patient management.

## 1. Introduction

Lung cancer is one of the most common and deadly diseases worldwide, accounting for 18.0% of cancer-related deaths [1]. Among them, small cell lung cancer (SCLC) is known for its poor prognosis, with median overall survival (OS) of 25–30 months in limited stage (LS) and 8–13 months in extensive stage (ES) [2,3]. The National Comprehensive Cancer Network guidelines historically endorsed platinum plus etoposide as the standard treatment for patients with ES-SCLC. However, recently, immune checkpoint inhibitors (ICIs) such as atezolizumab or durvalumab have emerged as preferred regimens, often in combination with conventional chemotherapy agents [4,5,6]. In the IMpower133 study, the addition of atezolizumab, an anti-programmed death ligand 1 (PD-L1) monoclonal antibody, to the carboplatin plus etoposide regimen resulted in a significant survival gain, with OS increasing from 10.3 months to 12.3 months (*p* = 0.0154) [4]. In the CASPIAN study, adding durvalumab to the first-line chemotherapy regimen increased the median OS by approximately three months, with no significant difference in the incidence of grade 3 or higher adverse events compared to the standard therapy group [6]. With promising results from these large-scale randomized controlled trials, patients with SCLC can now benefit from immunotherapy.

The effectiveness of ICIs, derived from modulating the innate immune system to control tumors, is counterbalanced by the significant costs associated with immune-related adverse events (irAEs), which exhibit considerable variability in severity and timing [7,8]. ICIs have been extensively utilized in the treatment of certain cancers, such as melanoma, renal cell carcinoma, or non-small cell lung cancer, resulting in well-documented adverse events [9,10,11,12,13,14,15]. However, for SCLC, where the adoption of ICIs is relatively recent, the reporting of side effects is less comprehensive than for other malignancies. Furthermore, the unexpected nature of irAEs presents significant diagnostic and management challenges for clinicians, especially given the rarity and limited documentation of these occurrences in real-world settings. Moreover, SCLC, comprised neuroendocrine cells, uniquely reacts to ICIs by producing biologically active substances. This potentially causes paraneoplastic syndromes like the syndrome of inappropriate secretion of antidiuretic hormone or ectopic Cushing’s syndrome, further complicating the adverse effects profile [16,17]. This complexity underscores the uncertainty surrounding clinical management strategies for these adverse events. To address these challenges, our study aims to systematically review and synthesize published clinical evidence, focusing on case reports that detail rare and poorly documented adverse events associated with using ICIs in SCLC. This review seeks to provide a comprehensive overview of existing knowledge, thus facilitating a better understanding and managing these complex clinical scenarios.

## 2. Materials and Methods

### 2.1. Search Strategy and Selection Criteria

A systematic literature search was conducted to identify all available case reports on using ICIs for patients with SCLC, published until 31 December 2023. An uncommon adverse event is defined as one occurring in fewer than 1% of patients; this threshold has recently been extended to include even rarer occurrences below 0.1% or 0.01%, presenting significant challenges in defining such events among heterogeneous or small patient cohorts [18,19]. Given that exceedingly rare adverse events are typically documented only in case reports, our study focused exclusively on compiling such reports for patients with SCLC. This approach was designed to examine uncommon adverse events in this specific context thoroughly.

**Figure 1:**
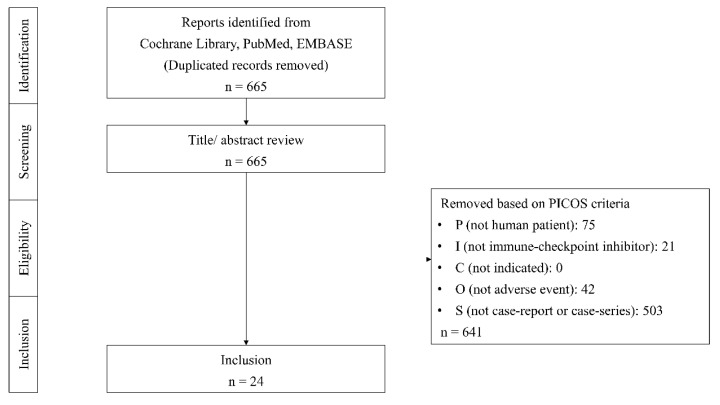
PRISMA diagram.

The first search query aimed to identify studies targeting patients with SCLC, while the second query encompassed all studies involving the administration of ICIs, specifically focusing on those presented in the form of case reports. The Cochrane Library, PubMed, and EMBASE electronic databases were utilized for literature searches, employing keywords such as (‘small cell lung cancer’/exp OR ‘small cell lung cancer’ OR (small AND (‘cell’/exp OR cell) AND (‘lung’/exp OR lung) AND (‘cancer’/exp OR cancer))) AND (‘immunotherapy’/exp OR immunotherapy OR ‘immune-checkpoint inhibitor’/exp OR ‘immune-checkpoint inhibitor’ OR ((‘immune’/exp OR immune) AND (‘checkpoint’/exp OR checkpoint) AND (‘inhibitor’/exp OR inhibitor)) OR ‘immune-checkpoint blockade’/exp OR ‘immune-checkpoint blockade’ OR ((‘immune’/exp OR immune) AND (‘checkpoint’/exp OR checkpoint) AND blockade)) AND ‘case report’/de AND (‘case report’/de OR ‘case study’/de). Additionally, manual searches of references were performed. The inclusion criteria were limited to English-language studies that met the Population, Intervention, Comparison, Outcomes, and Study (PICOS) criteria. The population (P) was defined as human subjects, with the intervention (I) focusing on the using ICIs. As case reports were the primary study design of interest, no specific comparison (C) was defined. The outcomes (O) were defined as irAEs, and the study (S) inclusion criterion encompassed only case studies (Figure 1).

### 2.2. Data Extraction

We systematically extracted key characteristics from the identified literature, documenting details including the name of the first author, publication year, journal of publication, patient demographics (age and sex), disease status, the specific ICI used, duration of treatment, and the features of irAEs. Regarding adverse events, we recorded the diagnosis of each adverse event, the symptoms manifested, the treatment methods employed for each symptom, and the outcomes of these treatments. Also, to minimize inter-researcher variability, a process of extracting characteristics from each report was repeated by each investigator, ensuring consistency in data extraction and analysis.

### 2.3. Quality Assessment

We performed quality assurance in all included studies (Supplementary Table S1). Owing to the distinct characteristics of case reports, we employed Pierson’s scoring system rather than traditional methodologies such as the Newcastle–Ottawa Scale [20,21]. This scoring system proposed an assessment framework for determining the credibility of a case report, comprising five elements: documentation, uniqueness, educational value, objectivity, and interpretation. In this scoring system, each category permits a range from 0 to 2 points, leading to a total maximum score of 10 points. Reports scoring between 9 and 10 are considered valuable contributions to the literature, indicating a high level of quality. For reports scoring between six and eight points, readers should exercise caution regarding their validity and clinical value, suggesting potential limitations or concerns, and reports scoring five or lower are considered of insufficient quality for publication. The average total score of the 24 studies was 9 points, with scores ranging from a minimum of 7 to a maximum of 10 points.

## 3. Results

### 3.1. Characteristics of the Included Studies

**Figure 2:**
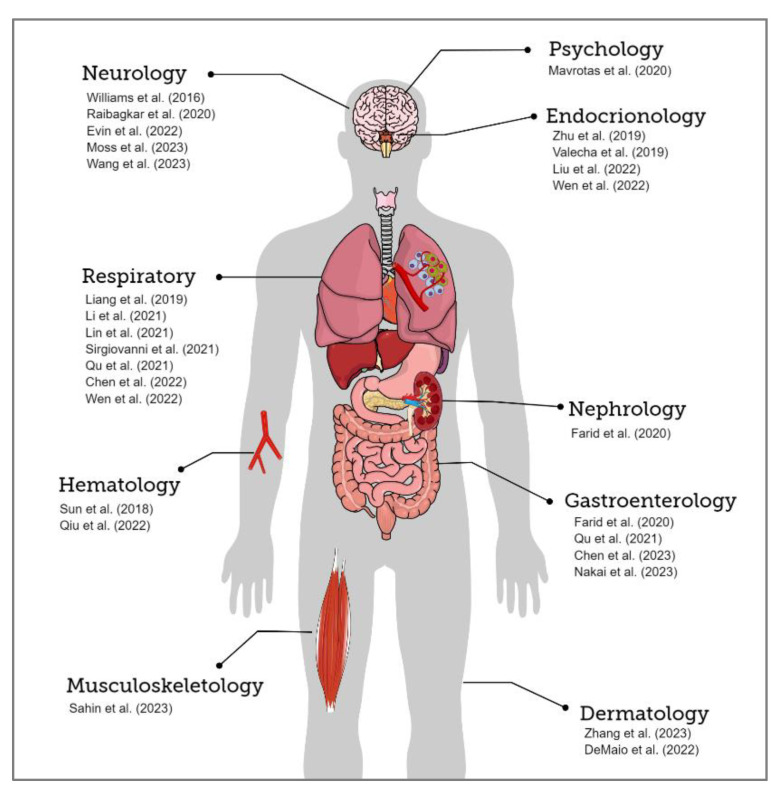
Case reports on immune-related adverse events caused by immune checkpoint inhibitors in patients with small cell lung cancer (first author and publication year); the occurrence of events by the system. References are listed starting from “Neurology” and proceeding in a counterclockwise order; Neurology: Williams et al. (2016) [22], Raibagkar et al. (2020) [23], Evin et al. (2022) [24], Moss et al. (2023) [25], Wang et al. (2023) [26]; Respiratory: Liang et al. (2019) [27], Li et al. (2021) [28], Lin et al. (2021) [29], Sirgiovanni et al. (2021) [30], Qu et al. (2021) [31], Chen et al. (2022) [32], Wen et al. (2022) [33]; Hematology: Sun et al. (2018) [34], Qiu et al. (2022) [35]; Musculoskeletology: Sahin et al. (2023) [36]; Dermatology: Zhang et al. (2023) [37], DeMaio et al. (2022) [38]; Gastroenterology: Farid et al. (2020) [39], Qu et al. (2021) [31], Chen et al. (2023) [40], Nakai et al. (2023) [41]; Nephrology: Farid et al. (2020) [39]; Endocrinology: Zhu et al. (2019) [42], Valecha et al. (2019) [43], Liu et al. (2022) [44], Wen et al. (2022) [33]; Psychology: Mavrotas et al. (2020) [45].

The final analysis included twenty-four studies. When categorized by ICI agents, there were six reports on atezolizumab, four on durvalumab, three on nivolumab monotherapy, six on the combination therapy of nivolumab, and one report each for other ICIs such as pembrolizumab, nofazinlimab, sintilimab, tislelizumab, and toripalimab. The characteristics of patients, types and dosages of ICI used, diagnosis of irAEs, and their respective onset times, as detailed in each report, are collectively summarized in Table 1. IrAEs were most frequently observed in the respiratory system when classified by the system, with seven cases reported. Following this, there were five cases of neurological events and four cases each of endocrinological and gastroenterological adverse events. Dermatological and hematological events were reported in two papers, and others were reported once per system (Figure 2).

**Table 1:** Characteristics of patients and immune-related adverse events.

First Author (Year)	Patent Characteristics			Immune Checkpoint Inhibitor		Immune-Related Adverse Event		
	Age	Sex	Disease Status *	Drug	Dosage	Diagnosis	System	Onset Time
Chen (2023) [40]	62	M	ES-SCLC	Atezolizumab	1200 mg	Elevation of Hepatitis B virus surface antibody	Gastroenterology	9 months
Evin (2022) [24]	64	M	ES-SCLC	Atezolizumab	1200 mg	PRES	Neurology	Several hours
Lin (2021) [29]	66	F	ES-SCLC	Atezolizumab	NA	Pleuritis	Respiratory	5 months
Nakai (2023) [41]	70	M	ES-SCLC	Atezolizumab	1200 mg	Colitis with CMV infection	Gastroenterology	7 months
Qiu (2022) [35]	64	M	ES-SCLC	Atezolizumab	NA	Thrombocytopenia, Anemia	Hematology	4.5 months
Sahin (2023) [36]	61	M	ES-SCLC	Atezolizumab	1200 mg	Myositis	Musculoskeletal	3.5 months
Moss (2023) [25]	72	M	ES-SCLC	Durvalumab	1500 mg	Limbic encephalitis	Neurology	10.5 months
Wang (2023) [26]	70	F	ES-SCLC	Durvalumab	NA	Paraneoplastic myelitis	Neurology	4.3 months
Wen (2022) [46]	65	M	ES-SCLC	Durvalumab	1000 mg	Pneumonitis, Autoimmune DM	Respiratory Endocrinology	7 months
Zhang (2023) [37]	60	M	ES-SCLC	Durvalumab	1000 mg	Onychopathy	Dermatologic	22 months
DeMaio (2022) [38]	69	F	ES-SCLC	Nivolumab	NA	Scleroderma-like syndrome	Dermatology	2.5 years
Raibagkar (2020) [23]	62	F	ES-SCLC	Nivolumab	NA	Worsening of paraneoplastic sensory neuropathy	Neurology	Few weeks
Zhu (2019) [42]	61	M	ES-SCLC	Nivolumab	3 mg/kg	Hypophysitis, IAD	Endocrinology	13 weeks
Liang (2019) [27]	44	F	ES-SCLC	Nivolumab + Atezolizumab	1200 mg, 240 mg	Pneumonitis	Respiratory	5 months
Farid (2020) [39]	Middle age	M	ES-SCLC	Nivolumab + Ipilimumab	3 mg/kg, 1 mg/kg	Hepatitis, Fanconi syndrome	Gastroenterology Nephrology	1 month
Mavrotas (2020) [45]	66	F	ES-SCLC	Nivolumab + Ipilimumab	NA	Acute mania	Psychiatric	3 months
Sirgiovanni (2021) [30]	44	M	ES-SCLC	Nivolumab + Ipilimumab	NA	Reactivation of tuberculosis	Respiratory	3 cycles of ICI
Valecha (2019) [43]	58	F	ES-SCLC	Nivolumab + Ipilimumab	NA	Hypophysitis	Endocrinology	2 cycles of ICI
Williams (2016) [22]	60	M	ES-SCLC	Nivolumab + Ipilimumab	1 mg/kg, 3 mg/kg	Autoimmune encephalitis	Neurology	4 days
Sun (2018) [34]	62	M	Unknown	Pembrolizumab	NA	Direct antiglobulin test-negative hemolytic anemia	Hematology	2 weeks
Liu (2022) [44]	43	M	Unknown	Nofazinilimab	NA	Autoimmune DM, DKA	Endocrinology	4.5 months
Li (2021) [28]	35	M	ES-SCLC	Sintilimab	200 mg	Hyperthermia, Pneumonitis	Respiratory	1 day
Chen (2022) [32]	67	M	LS-SCLC	Tislelizumab	200 mg	Steroid-refractory CIP	Respiratory	2.5 months
Qu (2021) [31]	72	M	ES-SCLC	Toripalimab	240 mg	Appendicitis, Biliary obstruction, Pneumonitis	Gastroenterology Respiratory	3 months

* The disease status denotes the condition of the disease at the onset of immune checkpoint inhibitor therapy. CIP, immune checkpoint inhibitor-related pneumonia; CMV, cytomegalovirus; DKA, diabetes ketoacidosis; DM, diabetes mellitus; ES-SCLC, extensive-stage small cell lung cancer; IAD, isolated ACTH deficiency; ICI, immune checkpoint inhibitor; PRES, posterior reversible encephalopathy syndrome.

### 3.2. Immune Checkpoint Inhibitor as Monotherapy

Most of the studies reported were on cases where it was used as monotherapy. Reports on atezolizumab totaled six, the highest for a single agent, with various adverse events observed across different systems. The adverse events observed after atezolizumab varied in duration, ranging from a few hours to as long as nine months, with the median onset time being six months. The shortest onset time reported was for a partial epileptic seizure occurring just hours after dosage, as described by Evin et al. [24]. This case, which developed symptoms such as right hemiplegia and high blood pressure even with anti-epileptic drugs, was diagnosed as a rare neurological phenomenon known as Posterior Reversible Encephalopathy Syndrome (PRES). There are some cases of PRES following the use of carboplatin or etoposide, but hardly any cases related to ICIs [47,48]. So far, only two documented cases of PRES, suspected to be linked to ICI therapy, have emerged after the second cycle of treatment in a phase I trial for retroperitoneal neuroendocrine carcinoma and after the fourth cycle in NSCLC, respectively [49,50]. In PRES, it is known that a hypertensive peak causes blood–brain barrier disruption and secondarily increases posterior circulation blood flow, leading to brain edema [51]. In this process, the addition of endothelial dysfunction contributes to the development of PRES. Subsequently, PRES can occur regardless of blood flow and pressure, indicating that the pathophysiology is poorly understood. Treatment has involved the use of antiepileptic drugs and corticosteroids to help alleviate the patient’s symptoms in this case. Additionally, the first reported adverse effect of atezolizumab monotherapy included biopsy-confirmed pleuritis and colitis accompanied by cytomegalovirus (CMV) infection. Lin et al. documented ICI-induced pleuritis in the absence of disease progression, representing the initial report of biopsy-confirmed pleuritis, notable for its onset several months after administration, underscoring its distinctiveness [29]. As is generally known, pneumonitis, a change in the lung parenchyma, is a common adverse event associated with ICIs; however, the effects on the pleura and pleural space are seldom reported [52]. Notably, this study is the first to verify the occurrence of pleuritis following the use of atezolizumab through histological examination. Nakai et al. [41] described the first case of ICI-induced colitis complicated by concurrent CMV infection, leading to colectomy followed by ileostomy due to colon perforation. Qiu et al. reported a case of severe thrombocytopenia and anemia occurring after 4.5 months of atezolizumab, which proved refractory to various treatments, including injection of intravenous immunoglobulin (IVIG), thrombopoietin, and plasma exchange and resulted in the expiration of the patient [35]. This represents the first documented case of refractory immune-related thrombocytopenia and anemia occurring simultaneously, notable for its occurrence in SCLC, while previous cases were observed in patients with NSCLC and melanoma [53,54]. Chen et al. reported an unusual increase in hepatitis B surface antibody (HBs Ab) in a 62-year-old patient with brain metastasis who was previously HBsAg-negative [32]. This patient was asymptomatic, and it was the first report of a continuous rise in HBs Ab levels following PD-L1 therapy. They proposed that this irAE is associated with the activation of both CD4+ and CD8+ T cells and might provide a solution to the insufficient production of protective antibodies post-vaccination [55,56]. In a case reported by Sahin et al., a patient experienced muscle weakness and dysphagia 3.5 months after receiving atezolizumab, accompanied by an increase in creatine phosphokinase [36]. Symptom relief was achieved through steroid administration, leading to a diagnosis of immune-related myositis. Compared to most cases where the mean time of onset of myositis is around one month, this case was reported as a late onset [46].

Subsequently, durvalumab-associated adverse events were the second most frequently reported when used as monotherapy. Durvalumab received approval from the United States Food and Drug Administration (FDA) for the treatment of SCLC in March 2020, which is more recent than other medications [57]. Most irAEs with durvalumab occurred several months after administration, with no cases demonstrating an exceptionally rapid onset. Two cases of neurological complications have been reported, one by Moss and another by Wang et al., where limbic encephalitis and paraneoplastic myelitis were documented, respectively [25,26]. A patient diagnosed with limbic encephalitis displayed seizure-like symptoms such as confusion, impaired balance, and difficulty walking [25]. Four days following the symptom onset, a magnetic resonance imaging (MRI) scan revealed an increased signal and volume in the left temporal lobe and hippocampus on T2 and FLAIR sequences, without the enhancement for the gadolinium contrast enhancement image, which contributed to the diagnosis. The patient underwent plasma exchange, and symptoms took over a year to fully resolve. Paraneoplastic myelitis, as reported by Wang et al., is typically a rare event occurring in less than 1% of cases [26]. This represents the first reported instance of durvalumab-related paraneoplastic neurologic syndrome. The patient experienced numbness and weakness in the lower limbs, requiring treatment with high doses of steroids, IVIG, and plasmapheresis. Zhang et al. reported ICI-induced onychopathy, characterized by pigmentation and thickening of the nails, as well as skin peeling and scaling on the hands, prompting the administration of anti-fungal medication [37]. While skin and nail peeling are common, these symptoms are rare, representing a novel manifestation in patient with lung cancer treated with durvalumab. Additionally, Wen et al. documented a case of concurrent ICI-induced pneumonitis and autoimmune diabetes mellitus (DM), marking the first instance of these two irAEs occurring simultaneously in a single patient [33]. Diagnosing ICI-induced pneumonitis is challenging due to its non-specific symptoms and imaging findings, necessitating its consideration as a diagnosis of exclusion. Additionally, ICI-induced autoimmune DM, displaying characteristics typical of both classic type 1 DM and fulminant diabetes, is considered a particularly rare subtype. The concurrent diagnosis and treatment of both conditions in a single patient are reported to be exceedingly rare and complex. As both conditions are life-threatening, early recognition and intervention are crucial.

Nivolumab, frequently associated with adverse effects as a monotherapy, is not currently a first-line treatment for SCLC. The FDA approval for relapsed SCLC was withdrawn due to insufficient survival benefits. It is only prescribed to patients with a chemotherapy-free interval of less than six months [58,59]. Nivolumab is recognized for causing unique immune-mediated adverse events, necessitating vigilant monitoring. The cases reporting adverse events of nivolumab monotherapy totaled three, ranging from a few weeks to 2.5 years from drug initiation to the occurrence of adverse events. In the report by Raibagkar et al., numbness in the extremities of the patient occurred a few weeks after drug initiation, accompanied by nonspecific gastrointestinal symptoms such as indigestion and diarrhea [23]. The patient was diagnosed with worsening paraneoplastic sensory neuropathy later and treated with IVIG and methylprednisolone, but showed no symptom improvement and remained wheelchair-bound. In a report by Zhu et al., after nine months of nivolumab, the patient developed anorexia, vomiting, and fatigue, leading to an adrenal crisis [42]. The laboratory tests showed low serum ACTH and hypothyroidism, indicating hypophysitis initially diagnosed as isolated ACTH deficiency. The patient subsequently underwent hormone replacement therapy. In contrast, in the report by DeMaio et al., adverse events occurred very late, approximately 2.5 years after drug initiation, and were diagnosed as an immunotherapy-induced scleroderma-like syndrome [38]. The patient experienced skin tightening and dysphagia and developed sclerotic plaques across the chest, arms, abdomen, and back, resulting in a restricted range of motion. An improvement in symptoms was reported after prolonged treatment with various therapies, including UVA, UVB, IVIG, and prednisone.

### 3.3. Combination Therapy with Other Immune Checkpoint Inhibitors

Since 2018, various medications have sequentially received FDA approval and have demonstrated efficacy as monotherapies, particularly in instances where conventional chemotherapy is ineffective, thus establishing themselves as viable treatment options. While Nivolumab is often used as a monotherapy, it is also frequently employed in combination with other ICIs. In the studies we analyzed, one case featured the combination of nivolumab and atezolizumab as a doublet therapy, whereas five cases involved the concurrent use with ipilimumab, a cytotoxic T-lymphocyte-associated protein 4 (CTLA-4) inhibitor [60,61].

Liang et al. reported pneumonitis after 4.5 months of combination therapy with atezolizumab [27]. The case represented the initial examples of severe pneumonitis observed after the sequential administration of atezolizumab and nivolumab, characterized by steroid-refractory pneumonitis. In a similar case, severe pneumonitis and myocarditis were reported following the administration of nivolumab after atezolizumab monotherapy; however, this instance occurred in a patient with lung squamous cell carcinoma [62].

The combination of nivolumab and ipilimumab has been associated with various irAEs, such as tuberculosis (TB) reactivation, Fanconi syndrome, acute mania, and autoimmune hypophysitis, typically reported within one month of usage. Sirgiovanni et al. reported TB reactivation in an asymptomatic patient, diagnosed through radiological and laboratory findings, with subsequent recovery following standard anti-TB medication [30]. Farid et al. reported Fanconi syndrome alongside immune-related hepatitis, representing a novel case despite renal injuries like acute kidney injury being commonly reported previously [39]. The patient suffered from metabolic acidosis and hypophosphatemia and improved after receiving treatment with intravenous bicarbonate and high-dose steroids. A case of acute mania reported by Mavrotas et al. occurred in an elderly female patient with no psychiatric history, exhibiting extensive symmetric leukoencephalopathy on MRI, suggesting an unusually rapid change and resistance to treatment [45]. She initially exhibited symptoms such as pyrexia, agitation, and confusion, and despite the administration of antipsychotic drugs, later displayed hostility, irritability, and persecutory delusions. The patient received additional treatment with zuclopenthixol acetate and lithium, leading to an improvement in symptoms. In autoimmune hypophysitis reported by Valecha et al., while it is frequently observed with the use of ipilimumab alone, they reported how the mechanism differs when both drugs are used together [43]. The patient exhibited symptoms typical of hypophysitis, including headache, nausea, vomiting, and decreased oral intake. Gadolinium-enhanced MRI of the brain revealed pituitary enlargement, and the administration of corticosteroids led to a rapid improvement in symptoms. Hypophysitis can occur with both ipilimumab and nivolumab. However, the incidence varies significantly between the two drugs, which is known to be quite rare when using nivolumab alone, occurring in less than 1% of cases [63,64,65]. However, a study by Wolchok et al. on patients with melanoma shows that combining drugs with distinct mechanisms results in significantly more severe side effects, notably increasing the incidence of hypophysitis to approximately 13% compared to monotherapy [66].

### 3.4. Immune-Related Adverse Events in Randomized Clinical Trials

Table 2 summarizes the major adverse events and their incidence rates in landmark randomized clinical trials targeting SCLC.

The IFCT-1603 study explored second-line treatments for recurrent SCLC by comparing a group receiving atezolizumab and conventional chemotherapy [67]. During the 13.7 month follow-up period, the atezolizumab group experienced irAEs such as hepatitis, colitis, arthralgia, and dysthyroidism; these manifestations were mild, graded as 1–2, and infrequently occurred, with an incidence of less than 5.0%. In the IMpower 133 study, atezolizumab was also the primary medication under investigation, distinct from earlier studies by evaluating its impact when combined with chemotherapy as a first-line treatment [4,5]. The irAEs were reported in 79 patients (39.9%) in the atezolizumab group compared to 48 patients (24.5%) in the placebo group, with rash and hypothyroidism being the most prevalent. Additionally, 4 patients (2.0%) in the atezolizumab group experienced grade 5 AEs, in contrast to 11 patients (5.6%) in the placebo group. Excluding hematologic adverse events, significant grade 3 or higher irAEs in the atezolizumab group included hepatitis, rash, and colitis. Rare side effects observed included Guillain–Barre syndrome, rhabdomyolysis, hypophysitis, and pancreatitis.

In the CASPIAN study, the impact of adding durvalumab to platinum-etoposide was evaluated [6]. Among the participants, 52 (20%) of the 265 patients treated with durvalumab plus platinum-etoposide experienced irAEs, compared to only seven (3%) of the 266 patients treated with platinum-etoposide alone. Although the majority of AEs were mild, with grade 1–2, 5% of individuals in the durvalumab group experienced severe side effects of grade 3 or higher, including one fatality due to hepatotoxicity, with thyroid dysfunction being the most frequently reported irAE. Additionally, less than 1% of the participants experienced rare side effects, with one individual each reporting adrenal insufficiency and pancreatic events.

In the CheckMate 331 trial, where nivolumab was used as a monotherapy, endocrine adverse effects were the most common, similarly to other ICIs [59]. In the CheckMate 451 and 032 trials, where nivolumab was combined with ipilimumab, both studies reported higher incidences of adverse events of any grade and grade 3 or 4 in the combination arms [68,69]. Notably, CheckMate 451 experienced seven treatment-related fatalities in the combination arm due to complications such as rhabdomyolysis, myocarditis, hepatic failure, limbic encephalopathy, myasthenia gravis, encephalitis, and immune colitis leading to bowel perforation, bacterial peritonitis, sepsis, and end-organ failure. In the CheckMate 032 trial, similarly to other studies, the most frequent adverse events included skin reactions, hematologic disturbances, and gastrointestinal toxicity. However, severe events led to fatalities, with one death in the monotherapy group and three in the combined therapy group attributed to hepatitis, pneumonitis, and encephalitis, respectively. Although a direct comparison may not be feasible, clinical trial data suggest that severe adverse events occur more commonly in the combination therapy group.

**Table 2:** Summary of clinical trials presenting immune-related adverse events for patients with extensive-stage or recurrent small cell lung cancer.

Clinical Trial	Phase	Target Group	Medication	Intervention	Immune-Related Adverse Event in Immune Checkpoint Inhibitor Group
NCT03059667 (IFCT-1603) [67]	II	Recurrent SCLC	Atezolizumab	Atezolizumab Chemotherapy (re-induction of carboplatin-etoposide doublet or second-line oral or intravenous topotecan)	Arthralgia (three patients (6.3%), two grade 1 and one grade 2) Hepatitis (two patients (4.2%), one grade 1 and one grade 2) Colitis (two patients (4.2%), both grade 1) Dysthyroidism (two patients (4.2%), one grade 1 hyperthyroidism, one hypothyroidism, grades 1 and 2, respectively)
NCT02763579 (IMpower 133) [4,5]	I/III	ES-SCLC	Atezolizumab	Atezolizumab + Carboplatin-etoposide Placebo + Carboplatin-etoposide	Rash (All grades, 18.7%; Grade 3–4, 2.0%) Hypothyroidism (All grades, 12.6%; Grade 3–4, 0.0%) Hepatitis (All grades 7.1%; Grade 3–4, 1.5%) Dysthyroidism (All grades 6.1%; Grade 3–4, 0.0%) Colitis (All grades 1.5%; Grade 3–4, 1.0%)
NCT03043872 (CASPIAN study) [6]	III	ES-SCLC	Durvalumab	Durvalumab + EP EP alone	Dysthyroidism (All grades, 14.0%; Grade 3–4, 0.0%) Pneumonitis (All grades, 3.0%; Grade 3–4, 1.0%) Hepatic event (All grades, 3.0%; Grade 3–4, 2.0%)
NCT02481830 (CheckMate 331) [59]	III	Recurrent SCLC	Nivolumab	Nivolumab (biweekly) Chemotherapy (topotecan or amrubicin)	Endocrine (All grades, 11.7%; Grade 3–4, 0.7%) Skin (All grades, 11.3%; Grade 3–4, 0.4%) Gastrointestinal (All grades, 7.1%; Grade 3–4, 1.1%) Hepatic (All grades, 4.6%; Grade 3–4, 2.5%) Pulmonary (All grades, 4.6%; Grade 3–4, 1.4%) Hypersensitivity (All grades, 4.3%; Grade 3–4, 0.0%) Renal (All grades, 2.1%; Grade 3–4, 0.4%) Treatment-related death in two patients (0.7%)
NCT02538666 (CheckMate 451) [69]	III	ES-SCLC	Nivolumab Ipilimumab	(1:1:1 randomization) Nivolumab + Ipilimumab Nivolumab alone (biweekly) Placebo	Any-grade and grade 3–4 treatment-related adverse events − 85.6% and 52.2% of the combination arm − 60.9% and 11.5% of the nivolumab arm − 50.2% and 8.4% of the placebo arm
NCT01928394 (CheckMate 032) [68]	I/II	Recurrent SCLC	Nivolumab Ipilimumab	Nivolumab + Ipilimumab Nivolumab alone	Any grade treatment related adverse events: 53.7% (nivolumab alone) vs. 68.8% (nivolumab + ipilimumab) Grade 3–4 treatment related adverse events: 2.9% (nivolumab alone) vs. 37.5% (nivolumab + ipilimumab)
NCT03066778 (KEYNOTE-604) [70]	III	ES-SCLC	Pembrolizumab	Pembrolizumab + EP Saline placebo + EP	Grade 3 irAEs: 7.2% (Pembrolizumab) vs. 0.9% (Placebo) No grade 4 or 5 ir-AEs in the pembrolizumab plus EP group

ES-SCLC, extensive-stage SCLC; EP, etoposide and platinum; ir-AE, immune-related adverse event; SCLC, small cell lung cancer.

## 4. Discussion

Immunotherapeutic agents targeting SCLC have been the subject of many clinical trials from first-line to maintenance, second-line, and beyond, showing promising results [4,6,59,67,68,69,70]. Ipilimumab, atezolizumab, durvalumab, pembrolizumab, and nivolumab are among the most commonly used ICIs in SCLC. Adverse events reported in clinical trials for these drugs have been well documented. In particular, PD-1/PD-L1 inhibitors have shown higher tolerance compared to CTLA-4 inhibitors [71]. Dermatologic events, colitis, and hepatitis are more frequent with CTLA-4 inhibitors, while pneumonitis, thyroid dysfunction, and rheumatologic issues are more common with PD-1/PD-L1 inhibitors. Current evidence from both preclinical and clinical studies has revealed that the therapeutic mechanisms associated with these treatments align with the pathways involved in the onset of autoimmune-related adverse effects [72,73,74,75]. Therefore, it has been established that the emergence of such side effects is unavoidable. However, unlike the predictable adverse effects associated with conventional cytotoxic chemotherapy agents, these immunologic side effects typically arise in an unforeseen manner, often presenting significant challenges for medical practitioners.

Adverse events induced by these agents occur across various cancer types, and for SCLC, certain researchers have also postulated such a hypothesis: the neuroendocrine nature of SCLC may lead to distinct adverse events, with neuromuscular toxicity being more prevalent in compared to NSCLC, as confirmed by meta-analysis [71]. Additionally, our study was limited to a collection of special cases, precluding statistical analysis; however, numerically, neurologic events were the most frequently reported, with five instances following adverse effects in the respiratory system. Furthermore, as the use of routine-based immunotherapy increases exponentially, previously overlooked or unexplained adverse events are recurring more frequently, potentially associated with checkpoint inhibitor therapy [76].

In this study, the significance lies in gathering and reporting these unique cases, aiming to facilitate prompt and accurate diagnosis and the development of treatment strategies through sharing cases. Among the included studies, there are instances where the reported side effects were documented for the first time in cases of lung cancer or SCLC. Zhang et al. reported onychopathy, documented for the first time in lung cancer patients receiving durvalumab, presenting predominantly with nail thickening and pigmentation rather than typical symptoms like nail detachment and redness, observed after pembrolizumab use [37,77]. Fanconi syndrome, reported by Farid et al., is also noteworthy for being the first observed in a patient with SCLC, presenting a distinct pathophysiology involving the loss of tolerance against endogenous kidney antigens [39,78]. Early recognition and prompt treatment are emphasized for acute interstitial nephritis, especially considering higher and earlier occurrence rates with combined therapy, as demonstrated in CheckMate 067, highlighting the need for extra caution [79]. Additionally, some cases have demonstrated the occurrence of multi-systemic adverse events in a single patient. Wen et al. documented the initial occurrence of simultaneous pneumonitis and autoimmune DM [33]. Qu et al. observed a series of adverse events, including appendicitis, biliary obstruction, and pneumonitis, manifesting sequentially in a single patient [31]. Cases of rare events not only present diagnostic challenges but also complicate treatment, as evidenced by a report by Nakai et al. on colitis combining CMV infection [41]. They highlight the importance of early initiation of antiviral therapy to mitigate the risk of perforation while awaiting tissue confirmation. In cases resistant to corticosteroid treatment, cautious dose escalation is advocated due to the heightened susceptibility to superimposed CMV infection [80]. High-dose steroids are the first-line treatment for most irAEs, but refractory cases with life-threatening situations may necessitate plasma exchange or IVIG administration, as seen in our studies [23,25,26,81,82].

There may be debates regarding the continuation of ICI agents in the presence of irAEs. Practical guidelines from the European Society for Medical Oncology and the American Society of Clinical Oncology group state permanent discontinuation of ICIs for patients with severe adverse events, except for endocrine cases. This exception is warranted as those with endocrine issues often require lifelong hormone replacement therapy, allowing continued ICI use alongside hormone supplementation [83,84,85]. Despite considerable efforts by investigators to develop biomarkers for predicting irAEs, a reliable and definite indicator remains elusive to date [86]. Nevertheless, with ongoing research aimed at creating tools that improve predictions by integrating multiple indicators, it is anticipated that forthcoming research will enhance predictive precision through more comprehensive and sophisticated studies [87].

## 5. Conclusions

Immunotherapy has introduced a spectrum of adverse events characterized by various onset times and unexpected symptoms. As the use of ICIs expands across different tumor types and stages, rare adverse events present significant diagnostic challenges. These require comprehensive evaluations and collaborative assessments to ensure timely diagnosis and improved patient outcomes. This review highlights the importance of recognizing these uncommon irAEs and underscores the need to incorporate biomarkers and innovative diagnostic technologies in guiding future therapeutic approaches.

## Data Availability

Data supporting the findings of this study are available upon request from the corresponding author.

## Supplementary Materials

The following supporting information can be downloaded at: https://www.mdpi.com/article/10.3390/cancers16101896/s1, Table S1: Quality assurance of included studies.
