# Supplementary material for: Uncommon Adverse Events of Immune Checkpoint Inhibitors in Small Cell Lung Cancer: A Systematic Review of Case Reports
Source: Cancers (Basel). 2024 May 16;16(10):1896. doi: 10.3390/cancers16101896 (PMC11119772; doi:10.3390/cancers16101896)
Supplement: Supplementary file 1 [file cancers-16-01896-s001.zip › cancers-2978463-supplementary.pdf]

Supplementary Table S1 Quality assurance of included studies

First Author (Year)	Source	Quality of case report					
		Documentation	Uniqueness	Educational value	Objectivity	Interpretation	Total
Chen (2023)	*Front. Oncol*	2	1	1	2	2	8
Evin (2022)	*Diagnostics (Basel)*	1	1	2	2	2	8
Lin (2021)	*Am J Case Rep*	1	1	2	2	2	8
Nakai (2023)	*Respir Med Case Rep*	2	1	2	2	2	9
Qiu (2022)	*Transl Lung Cancer Res*	2	2	2	2	2	10
Sahin (2023)	*J Oncol Pharm Pract*	2	1	1	2	2	8
Moss (2023)	*Front. Immunol*	2	1	2	2	2	9
Wang (2023)	*Invest New Drug*	2	2	2	2	2	10
Wen (2022)	*Medicine (Baltimore)*	1	2	2	2	2	9
Zhang (2023)	*Clin Cosmet Investig Dermatol*	2	2	2	2	2	10
DeMaio (2022)	*JAAD Case Rep*	2	1	2	2	2	9
Raibagkar (2020)	*J Neuroimmunol*	2	2	2	2	2	10
Zhu (2019)	*Onco Targets Ther*	1	2	1	1	2	7
Liang (2019)	*Front. Oncol*	1	2	2	2	2	9
Farid (2020)	*Cureus*	2	2	2	2	2	10
Mavrotas (2020)	*Prog Neurol Psychiatry*	2	1	1	2	2	8
Sirgiovanni (2021)	*Thorac Cancer*	2	2	2	2	2	10
Valecha (2019)	*J Oncol Pharm Pract*	2	2	2	2	2	10
Williams (2016)	*JAMA Neurol*	2	2	2	2	2	10
Sun (2018)	*J Immunother*	2	2	2	2	2	10
Liu (2022)	*Transl Oncol*	2	1	1	2	1	7
Li (2021)	*Thorac Cancer*	2	2	2	2	2	10
Chen (2022)	*Transl Cancer Res*	2	2	2	2	2	10
Qu (2021)	*Ann Palliat Med*	2	1	2	2	2	9

Listed by immune-checkpoint inhibitors, and within each agent, listed in alphabetical order of the first author's name.
